# Round the Hospitals

**Published:** 1917-02-17

**Authors:** 


### ROUND THE HOSPITALS.

The vacancy at St. Mary's Hospital, Paddington, caused by the appointment of Miss Ruth E. Darbyshire, R.R.C., to the important post of chief lady superintendent of Lady Minto's Indian Nursing Association, has been filled by the appointment of Miss K. F. Muriel Jackson. Miss Jackson has shown from the outset of her nursing career a commendable desire to learn, to gain experience, and to qualify herself to occupy one of the higher positions in the British nursing world. Trained at St. George's Hospital, S.W., 1903-7, on the completion of her training she took a course in midwifery at the General Lying-in Hospital, York Road. In 1907 she returned to St. George's Hospital as ward sister, where she served until 1910, when she became housekeeping sister at the same hospital for a year. In November 1911 she obtained the appointment of assistant matron to the Royal Infirmary, Liverpool—a splendid field for good work, where many experiences of the highest value are always to be gained. In April 1913 she left Liverpool on being appointed matron to the Warneford General Hospital, Leamington, a post which she relinquishes on taking up her duties at St. Mary's Hospital, Paddington. All who know her will wish Miss Muriel Jackson a happy, prosperous, useful, and successful career at St. Mary's Hospital, where there is much good work to be done and a wide field for the introduction of modern ideas and the highest standards. Miss Jackson has won her way by steady and continuous work. Her future will be followed with interest by all with whom she has been associated during her nursing career.

(*Continued on p.* 412.)

### ROUND THE HOSPITALS—(*continued from p.* 410).

The meeting in support of the Royal College of Nursing in Belfast, which was attended by Miss Cox-Davies and Miss Rundle, was one of the most stimulating, helpful, and pleasant meetings yet held. It was a large and representative meeting. It included most, if not all, the more active spirits interested in hospitals and nursing amongst the workers in the North of Ireland. Everywhere there was evidence of the presence of a spirit of welcome to the representatives of the College, and everyone seemed anxious to do anything in their power to promote the work of the College and to secure a share in it for Ireland. Miss Cox-Davies is a very tactful, able, and persuasive speaker. She naturally won the attention of her audience, and she seems on a fair way to become the Demosthenes of the modern nursing world. It was most cheering to note how quick the Irish nurses were to realise the professional advantages to be gained through the College and its future prospects. Again the same questions are everywhere engaging the thoughts of nurses; thus the problem of the small and special hospitals with its possible solution by amalgamation, and post-graduate courses, and the prospect of lay representation on the present Council—viz., two out of the forty-five members, one being the chairman—were raised and fully dealt with. A new suggestion made by a practitioner was that geographical areas might be represented on the Council. Professor Lindsey, the chairman, spoke encouragingly of the College scheme, wished the Council good luck in its future developments, and congratulated the nurses on the prospects it offered them and their profession. Altogether the meeting was a remarkable success. No words can exaggerate the appreciation felt towards Miss Bostock, the matron of the Royal Victoria Infirmary, Belfast, for her attractive hospitality and friendly and generous help.

